# The inverted CD4/CD8 ratio and associated parameters in 66-year-old individuals: the Swedish HEXA immune study

**DOI:** 10.1007/s11357-012-9400-3

**Published:** 2012-03-14

**Authors:** J. Strindhall, M. Skog, J. Ernerudh, M. Bengner, S. Löfgren, A. Matussek, B. O. Nilsson, A. Wikby

**Affiliations:** 1 School of Health Sciences, Department of Natural Science and Biomedicine, Jönköping University, Box 1026, 551 11 Jönköping, Sweden; 2 Institution of Clinical and Experimental Medicine, Division of Cell Biology, Linköping University, Linköping, Sweden; 3 Division of Clinical Immunology, Department of Molecular and Clinical Medicine, Faculty of Health Sciences, Linköping University, Linköping, Sweden; 4 Department of Infectious Diseases, Ryhov Hospital, 551 85 Jönköping, Sweden; 5 Department of Laboratory Medicine, Clinical Microbiology, Ryhov County Hospital, 551 85 Jönköping, Sweden

**Keywords:** Immune profile, Immunosenescence, T-lymphocytes, Age, Gender

## Abstract

The Swedish OCTO and NONA immune longitudinal studies were able to identify and confirm an immune risk profile (IRP) predictive of an increased 2-year mortality in very old individuals, 86–94 years of age. The IRP, was associated with persistent cytomegalovirus infection and characterized by inverted CD4/CD8 ratio and related to expansion of terminally differentiated effector memory T cells (TEMRA phenotype). In the present HEXA immune longitudinal study, we have examined a younger group of elderly individuals (*n* = 424, 66 years of age) in a population-based sample in the community of Jönköping, Sweden, to examine the relevance of findings previously demonstrated in the very old. Immunological monitoring that was conducted included T cell subsets and CMV-IgG and CMV-IgM serology. The result showed a prevalence of 15 % of individuals with an inverted CD4/CD8 ratio, which was associated with seropositivity to cytomegalovirus and increases in the level of TEMRA cells. The proportion of individuals with an inverted CD4/CD8 ratio was significantly higher in men whereas the numbers of CD3+CD4+ cells were significantly higher in women. In conclusion, these findings are very similar to those previously found by us in the Swedish longitudinal studies, suggesting that an immune profile previously identified in the very old also exists in the present sample of hexagenerians. Therefore, it will be important to examine clinical parameters, including morbidity and mortality, to assess whether the immune profile also is a risk profile associated with higher mortality in this sample of hexagenerians.

## Introduction

The mean lifespan for humans in industrialized countries is steadily increasing, and the very old constitute the fastest growing age segment with compromised health and increased need for advanced health care. Dysfunction of the immune system is commonly assumed to be responsible for the increased susceptibility to infectious disease and reduced response to vaccination in old people (Pawelec et al. 2010).

In the Swedish OCTO Immune Longitudinal study (referred to here as OCTO), we identified an immune risk profile (IRP) among very old individuals (85+) selected on the basis of individuals being at good health. Cluster analysis of immune data that was composed of the proliferative response to the mitogen Concanavalin A (ConA), and the percentages of CD3, CD4, CD8, and CD19 positive cells, identified an IRP predictive of 2-year mortality. The IRP cluster was characterized by high levels of CD8 and low levels of CD4 and CD19 together with a decreased response to ConA (Ferguson et al. 1995). This combination of immune parameters was later designated as the immune risk profile (Pawelec et al. 2001). Furthermore, the studies have shown that persistent cytomegalovirus (CMV) infection as well as the accumulation of lately differentiated CD8+CD28−CD27− cells are likely to be associated with the IRP (Olsson et al. 2000; Reker-Hadrup et al. 2006).

The results from the OCTO provided the background to the NONA immune longitudinal study (referred to here as NONA) in a sample of very old individuals (86+) without excluding individuals due to compromised health (Wikby et al. 2002). Here, IRP was identified using the inverted CD4/CD8 ratio, since this marker was found to be significantly associated with the risk profile. The NONA confirmed the findings obtained in the OCTO. Moreover, it showed that IRP was independent of the overall health status in the NONA sample, in which as much as 63 % comprised of frail individuals (Wikby et al. 2002; Nilsson et al. 2003).

In an attempt to expand these results to the whole lifespan of an adult, a retrospective study was conducted using another population-based study, Kristianstad Survey (KRIS), together with the OCTO and NONA studies (Wikby et al. 2008). Notably, the prevalence of a CD4/CD8 ratio less than 1 increased from about 8 % in age span of 20–59 years to about 16 % in ages between 60 and 94. The mortality rate in individuals with an inverted CD4/CD8 ratio was increased above the age of 60 years. In addition, the proportion of individuals with an inverted CD4/CD8 ratio was significantly higher in men than in women. Unfortunately, the KRIS study did not provide data on CMV infection or on lately differentiated CD8+CD28−CD27− cells that are associated with the IRP.

The aim of the present study was to further test the results obtained in the KRIS, to assess whether the IRP might be relevant among younger elderly similarity with previous findings in the very old. Here, we report data from a new Swedish HEXA immune longitudinal study of individuals with 66 years of age. The study investigates the prevalence of individuals with an inverted CD4/CD8 ratio as well as data on T and B cell subsets, CMV infection, and gender, which were previously found to be associated with an IRP in very old individuals.

## Materials and methods

### The Swedish HEXA study subjects

A population-based sample of 66-year-old individuals with no exclusion criteria formed the framework of this study. Census data collected from January 2009 was used to identify these hexagenerians (referred here as HEXA-1 individuals) permanently residing within the municipality of Jönköping, located in the south-central part of Sweden. From 1,310 66-year-old potential participants, 655 were randomly selected and invited. Blood samples were consecutively collected until receiving 424 participants which was previously decided based on power analysis. A non-proportional sampling procedure between men and women was used. Of the 424 participants, 227 were women (53.5 %) and 197 men (46.5 %). Every participant answered a questionnaire concerning diseases in the past or present, ongoing medication, exercise, and smoking.

In order to establish a deeper analysis of the immune profile that was obtained from the screening of individuals from HEXA-1, a new random selection of HEXA-1 individuals was performed. HEXA-1 participants were invited to HEXA-2 based on the results from HEXA-1. Fifty individuals with a CD4/CD8 ratio less than 1 and 101 individuals with a CD4/CD8 ratio more than 1.2 comprised the sample of HEXA-2.

### Blood and cell preparations from HEXA-1 and HEXA-2 individuals’

Venous blood samples were drawn at health care centers in the municipality of Jönköping. For HEXA-1 individuals, three EDTA vacutainer tubes (BD Biosciences, Stockholm Sweden) of 3 ml whole blood were drawn from each subject between 9 and 12 am and were analyzed within 24 h. One tube was used for flow cytometric analysis. The other two tubes were prepared by centrifugation at 1,500 × *g* for 10 min. Plasma was transferred to separate micro tubes (VWR International, Stockholm, Sweden) and stored at −70 °C for IgG and IgM serology analysis. For HEXA-2 individuals, one Na-heparin vacutainer tube of 4 ml was drawn. The blood samples were used for flow cytometric analysis.

### Flow cytometric analysis

In HEXA-1, a flow cytometry panel was used to define major lymphocyte populations. Data was acquired using a FACSCanto flow cytometer (BD Biosciences, San José, CA), and the Diva software (BD Biosciences) was used to calculate absolute numbers as well as percentages of T cell subsets. Briefly, 50 μl of whole blood was stained with 5 μl of an antibody cocktail containing the following antibodies: CD3 FITC, CD4 PE-Cy7, CD8 APC-Cy7, CD16 PE+CD56 PE, CD19 APC, and CD45 PerCP-Cy5.5 (BD Biosciences). After 15 min incubation in room temperature in the dark, 450 μl of BD FACS^TM^ lysing solution was added. The BD TrueCount™ tubes were used for accurate calculation of the lymphocyte numbers. For HEXA-2 individuals, an extended panel was used with fluorochome label antibodies against surface antigen on following lymphocyte subsets: CD3 APC H7, CD4 FITC, CD8 FITC, CD27 APC, CD28 PerCP-Cy5.5, and CCR7 PE (BD Biosciences).

### Cytomegalovirus serology

Plasma from all individuals in HEXA-1 was analyzed for IgG and IgM antibodies against CMV according to the manufacturer’s instructions (Zeus Scientific, Branchburg, NJ, USA). Absorbance was measured and an optical density (OD) ratio was calculated. OD values was interpreted as negative (<0.90), equivocal (0.91–1.09) and positive (>1.10).

### Data analysis

Statistical analyses were conducted by using SPSS 14. Student’s *t* test was used for comparison of independent groups. The chi-square test was used for analyzing the subgroups of CD4/CD8 ratio, CMV carrier status, and gender.

## Results

### Prevalence of individuals with an inverted CD4/CD8 ratio and frequency of T and B cell subsets for HEXA-1 individuals with CD4/CD8 ratio less than or greater than 1

Sixty-two individuals out of 424 (14.6 %) had a CD4/CD8 ratio less than 1. The percentages and numbers of T and B cells are shown in Table 1. Individuals with a CD4/CD8 ratio less than 1 showed a significant increase in the percentages and numbers of CD3+CD8+ cells and decreased percentages and numbers of CD3+CD4+ cells. Individuals with CD4/CD8 ratio less than 1 also showed a significant decrease in the percentage of CD19 B cells.

**Table 1:** Proportions (%) and numbers (per μl) of T and B cell subsets in 66-year-old individuals categorized by their CD4/CD8 ratio

Cell subset	CD4/CD8 <1	CD4/CD8 >1	*p*<
	*n* = 62	*n* = 362	
CD3 (%)	76.3 ± 1.2^a^	71.4 ± 0.5	0.001
CD4 (%)	30.6 ± 0.7	46.6 ± 0.4	0.001
CD8(%)	40.6 ± 1.1	20.7 ± 0.4	0.001
CD19 (%)	8.5 ± 0.7	10.7 ± 0.2	0.01
CD3 (cells/μl)	2,018 ± 85	1,542 ± 27	0.001
CD4 (cells/μl)	807 ± 36	1,004 ± 18	0.001
CD8 (cells/μl)	1,083 ± 54	451 ± 12	0.001
CD19 (cells/μl)	213 ± 18	232 ± 7	NS

^a^Mean ± SE

### Prevalence of CMV IgG and IgM antibodies and relation to CD4/CD8 ratio

Overall, 327 out of 424 (77 %) were CMV-IgG positive and 24 (5.6 %) were CMV-IgM positive. CMV-IgG was significantly more common in individuals with CD4/CD8 ratio less than 1, and for CMV-IgM, there was a tendency in the same direction (Table 2). In CMV-IgG positive individuals, the levels of CMV-IgG antibodies were significantly higher in those with a CD4/CD8 ratio less than 1 (3.3 ± 1.0, *n* = 14) compared to those with a ratio greater than 1 (3.0 ± 0.9 *n* = 271, *p* < 0.05).

**Table 2:** Chi-square analysis of relationships of cytomegalovirus IgG and IgM antibodies in 66-year-old individuals categorized by their CD4/CD8 ratio

CMV antibody status	CD4/CD8 <1	CD4/CD8 >1	*p*<
	*n* = 62	*n* = 362	
IgG			
+	56 (90.3)^a^	271 (74.9)	0.05
+/−^b^	0 (0.0)	2 (0.6)	
−	6 (9.7)	89 (24.6)	
IgM			
+	5 (8.1)	19 (5.2)	NS
+/−^b^	3 (4.8)	7 (1.9)	
−	54 (87.1)	336 (92.8)	

^a^No (% within CD4/CD8 subgroup)

^b^Equivocal

### Proportions and absolute numbers of T and B cell subsets for CMV-IgG positive and negative individuals

There were significant increases in the percentages and numbers of CD3+ and CD3+CD8+ cells and decreases in CD3+CD4+ cells in CMV-IgG positive individuals compared with negative individuals (Table 3). CMV-IgG positive individuals also showed a significantly lower CD4/CD8 ratio compared with the CMV-IgG negative individuals.

**Table 3:** Proportions (%) and numbers (per μl) of T and B cell subsets in 66-year-old CMV-IgG positive and negative individuals

Cell subset	CMV-IgG		*p*<
	Positive	Negative	
	*n* = 327	*n* = 95	
CD3 (%)	72.9 ± 0.5^a^	69.5 ± 1.0	0.01
CD4 (%)	43.4 ± 0.5	47.3 ± 0.4	0.01
CD 8 (%)	25.5 ± 0.6	17.2 ± 0.9	0.001
CD 19 (%)	10.2 ± 0.3	10.9 ± 0.5	NS
CD 3 (cells/μl)	1,697 ± 32	1,326 ± 39	0.001
CD 4 (cells/μl)	997 ± 20	897 ± 29	0.01
CD 8 (cells/μl)	608 ± 20	328 ± 19	0.001
CD 19 (cells/μl)	234 ± 8	214 ± 13	NS
CD4/CD8	2.12 ± 0.08	3.55 ± 0.22	0.001

^a^Mean ± SE

### Proportions and absolute numbers of T and B cell subsets for CMV-IgG positive and negative individuals with a CD4/CD 8 ratio less than 1

CMV-IgG positive individuals showed significantly higher numbers of CD3+, CD4+, and CD8+ cells as compared with negative individuals (Table 4). The CMV-IgG positive individuals also showed a lower CD4/CD8 ratio, although the difference between the groups did not reach statistical significance.

**Table 4:** Proportions (%) and numbers (per μl) of T and B cell subsets in 66-year-old CMV-IgG positive and negative individuals with CD4/CD8 ratio less than 1

Cell Subset	CMV-IgG		*p*<
	Positive	Negative	
	*n* = 56	*n* = 6	
CD3 (%)	75.9 ± 1.2^a^	80.1 ± 3.3	NS
CD4 (%)	30.3 ± 0.7	33.4 ± 1.5	NS
CD8 (%)	40.7 ± 2.1	40.2 ± 2.7	NS
CD19 (%)	8.7 ± 0.8	6.6 ± 0.9	NS
CD3 (cells/μl)	2,052 ± 93	1,702 ± 58	0.01
CD4 (cells/μl)	817 ± 40	710 ± 26	0.05
CD8 (cells/μl)	1,108 ± 59	852 ± 47	0.01
CD19 (cells/μl)	221 ± 20	139 ± 18	NS
CD4/CD8	0.77 ± 0.02	0.85 ± 0.05	NS

^a^Mean ± SE

### Proportions (%) of naïve and terminally differentiated effector memory CD3+CD8+CD28−CCR7−CD45RA+cells related to CD4/CD8 ratio and CMV-IgG status in HEXA-2 individuals

The percentage of terminally differentiated effector memory (TEMRA) cells in individuals with a CD4/CD8 ratio less than 1 was significantly higher than in those with a CD4/CD8 ratio greater than 1 (Table 5). For CMV-IgG positive individuals from the entire HEXA-2 sample, the percentage of TEMRA cells were significantly higher than in CMV-IgG negative individuals. The latter individuals also showed a significantly higher proportion of naïve cells. Individuals with a CD4/CD8 ratio less than 1 and those who were CMV-IgG positive showed significantly higher percentages of TEMRA cells compared to those who were CMV-IgG negative.

**Table 5:** The proportions (%) of naïve and terminally differentiated effector memory CD45RA+ T cells (TEMRA) among CD3+CD8+ cells in CD4/CD8 and CMV-IgG subgroups in HEXA 2 individuals

Subgroup	TEMRA (%)	*p*<	Naïve (%)	*p*<
CD4/CD8 (entire group)				
<1 (*n* = 50)	43.2 ± 2.6^a^	0.001	6.4 ± 1.1	0.001
>1 (*n* = 101)	22.9 ± 1.5		16.2 ± 1.2	
CMV-IgG (entire group)				
Positive (*n* = 118)	34.7 ± 1.6	0.001	10.8 ± 1.0	0.001
Negative (*n* = 31)	12.0 ± 1.6		20.0 ± 2.1	
CMV-IgG (CD4/CD8 <1 individuals)				
Positive (*n* = 46)	45.0 ± 2.6	0.05	5.9 ± 1.2	NS
Negative (*n* = 4)	22.6 ± 5.5		12.3 ± 4.3	

^a^Mean ± SE

### Proportions (%) and numbers (per ul) of T and B cell subsets, the CD4/CD8 ratio and CMV-IgG and IgM status by gender in HEXA-2 individuals

Females showed significantly higher percentages and numbers of CD3+CD4+ and CD19+ cells as well as numbers of CD3+ cells compared to males (Table 6). A CD4/CD8 ratio less than 1 was more common in males, although females showed a higher prevalence of CMV-IgG antibodies (NS).

**Table 6:** Proportions (%) and numbers of T and B cell subsets and the prevalence of individuals with inverted CD4/CD8 ratio and CMV carrier status by gender in 66-year-old individuals

Variable	Male	Female	*p*<
	*n* = 197	*n* = 227	
CD3 (%)	71.6 ± 0.7^a^	72.7 ± 0.6	NS
CD4 (%)	42.9 ± 0.7	45.4 ± 0.6	0.05
CD8 (%)	21.4 ± 0.8	23.2 ± 0.6	NS
CD19 (%)	9.5 ± 0.3	11.1 ± 0.3	0.01
CD3 (cells/μl)	1,523 ± 39	1,689 ± 38	0.01
CD4 (cells/μl)	891 ± 22	1,048 ± 25	0.001
CD8 (cells/μl)	536 ± 27	550 ± 21	NS
CD19 (cells/μl)	198 ± 8	257 ± 10	0.001
CD4/CD8 <1 (%)	18.8	11.0	0.05
CMV-IgG (%)	73.1	80.6	NS
CMV-IgM (%)	5.6	5.7	NS

^a^Mean ± SE

## Discussion

We investigated T and B cell subsets as well as CMV status in a 66-year-old Swedish population-based sample with a CD4/CD8 ratio less/greater than 1. The present study demonstrates that individuals with an inverted CD4/CD8 ratio reveal an immune profile consisting of high CD8 and low CD4 and CD19 percentages, associated with seropositivity to CMV as well as to significant increases in the number of lately differentiated CD8+CD28− cells. An IRP associated with increased mortality, consisting of high CD8 and low CD4 and CD19 percentages and poor proliferative response to ConA, was initially identified using a cluster analysis approach in octogenarian individuals participating in the Swedish OCTO Immune study (Ferguson et al. 1995). Subsequent studies of very old individuals participating in the Swedish OCTO and NONA immune studies indicated that this IRP could be defined using only the inverted CD4/CD8 ratio (Wikby et al. 1998, 2002). These findings were supported by a study showing that an inverted CD4/CD8 ratio predicted survival in a U.K. sample of elderly people (Huppert et al. 2003). Further, the presence of an inverted CD4/CD8 ratio in individuals above the age of 60 was also confirmed in the KRIS study of Swedish individuals in the age range of 20–79 years (Wikby et al. 2008). Strikingly, all these studies did show a prevalence of old individuals with an inverted CD4/CD8 ratio of 16 % which is close to the finding of 15 % in the present HEXA study. Thus, our findings demonstrate that 66-year-old individuals show an immune profile previously identified in the very old to be an IRP. It will be important to follow up immune system changes as well as clinical outcomes of these HEXA individuals to establish if the immune profile has the same relevance in younger old compared with older old individuals.

The present study also identified changes with increased levels of terminally differentiated CD8+ cells with a CD3+CD8+CD27−CD28−CD45RA+CCR7−perforin+phenotype (TEMRA) and a depletion of the proportion of CD8+CCR7+CD45RA+naïve cells to be associated with an inverted CD4/CD8 ratio along with IgG seropositivity to CMV. These results suggest that CMV might be a driving force in the generation of TEMRA CD8+ cells also in this age group. Evidence for a major impact of CMV in generating lately differentiated CD8+ cells was earlier demonstrated in the OCTO subjects by tetramer technology (Ouyang et al. 2003) and was also confirmed in the subsequent NONA immune study in very old individuals (Reker-Hadrup et al. 2006). Evidence has also been given that CD8+CD28− cells possess the characteristics of replicative senescence, including telomere shortening and apoptosis-resistance (Spaulding et al. 1999; Effros 2007). In addition, T cell-mediated immunity appears to be particularly susceptible to aging by a decrease in the number and diversity of naïve T cells dramatically decreased with the age-related thymic involution (Lang et al. 2011). These naïve cells have been reported to exhibit functional defects, including significantly shorter telomeres, a restricted receptor repertoire, reduced IL-2 production, and impaired differentiation into effector T cells (Lang et al. 2011). Consequently, their ability to mediate effective immune responses against new antigens might be decreased (Aspinall et al. 2010). The changes found in the T cell system of the present study therefore suggest that inversion of CD4/CD8 ratio is a marker of immunosenescence.

In our previous KRIS study (Wikby et al. 2008), we included data from a population-based sample in the age range of 20–79 years to examine the prevalence of individuals with an inverted CD4/CD8 ratio relative to age and gender. The percentage of individuals with an inverted CD4/CD8 ratio increased from about 8 % in the age range of 20–59 years to about 16 % in the age range of 60–94 and showed that the proportion of individuals with an inverted ratio was significantly higher in men whereas the numbers of CD3+CD4+ cells were found to be significantly higher in women, regardless of age, indicating that genetic or thymic factors, or both, might be of importance in balancing the relative numbers of CD3+CD4+ and CD3+CD8+ T cells in the individual. Interestingly, these data are strikingly similar to those found in the present study and are close to those presented by Amadori et al. (1995) and Clementi et al. (1999). The latter studies indicated that within a population of healthy blood donors, the CD4/CD8 ratio varies; with 5 % of individuals having values less than 1 and that as much as 57 % of the variation is under genetic control (Amadori et al. 1995). In the HEXA study, this would correspond to a finding of 21 individuals with an inverted CD4/CD8 ratio and since the prevalence of persistent CMV infection is 77 %, we would expect to find 16 of these 21 individuals to be CMV positive whereas five individuals would be CMV negative by random. This is in sharp contrast to the actual finding of 62 individuals with an inverted CD4/CD8 ratio, i.e., almost three times higher prevalence, with 56 being CMV positive and six being negative. Since the finding of six CMV negative individuals is close to what would be expected from a genetic point of view, it is plausible that an inverted CD4/CD8 ratio in these individuals is associated with genetic factors. This notion is also supported by the findings that CMV negative individuals with an inverted CD4/CD8 ratio did not show any T cell changes associated with CMV, while those being positive did. Our findings of 56 CMV-IgG positive individuals with an inverted CD4/CD8 ratio, however, seem to be far too many to be explained only by means of a normal genetic distribution. Instead, it seems plausible that a majority of these individuals have moved into the <1 category by decreases in their CD4/CD8 ratio. CMV infection seems to have had a major impact on recruiting additional individuals into this category by the generation of CD3+CD8+CD28− cell expansions, changes that we also identified previously in the very old (Wikby et al. 1998). Increases in the number of individuals being IgM seropositive as well as significant increases in the CMV-IgG antibody levels in individuals with a CD4/CD8 ratio less than 1 support the hypothesis that these expansions might be due to increased CMV reactivation and replication (Stowe et al. 2007). The clinical relevance in the measurement of CMV-IgG antibody levels in studies of immune system and aging was recently demonstrated by findings supporting an independent association between CMV IgG antibody level and 7-year mortality in community-living older adults (Strandberg et al. 2009). CMV was also associated with a significant increased risk for all-cause mortality in a U.S. nationally representative study (Simanek et al. 2011). The present study also supports that the changes described become important by the age of 65 (Saule et al. 2006; Wikby et al. 2008).

In conclusion, the results indicate that the immune profile, previously shown in octo- and nonagenerians to be associated with increased mortality (an IRP), also exists in the present sample of hexagenerians. Therefore, it will be important to examine morbidity and mortality to assess whether the profile also is a risk profile in this sample (an IRP) of hexagenerians.
